# Childhood Environment Influences Adrenarcheal Timing among First-Generation Bangladeshi Migrant Girls to the UK

**DOI:** 10.1371/journal.pone.0109200

**Published:** 2014-10-13

**Authors:** Lauren C. Houghton, Gillian D. Cooper, Mark Booth, Osul A. Chowdhury, Rebecca Troisi, Regina G. Ziegler, Hormuzd A. Katki, Robert N. Hoover, Gillian R. Bentley

**Affiliations:** 1 Division of Cancer Epidemiology and Genetics, National Cancer Institute, National Institutes of Health, Bethesda, Maryland, United States of America; 2 Department of Anthropology and Wolfson Research Institute for Health and Wellbeing, Durham University, Durham, United Kingdom; 3 School for Medicine, Pharmacy and Health, Durham University, Durham, United Kingdom; 4 Sylhet MAG Osmani Medical College, Sylhet, Bangladesh; London School of Hygiene and Tropical Medicine, United Kingdom

## Abstract

**Background:**

Adrenarche is a key early life event that marks middle childhood at approximately 7 years of age. Earlier work with British-Bangladeshi migrant women suggested that environmental conditions experienced before adrenarche influence adult reproductive function. We therefore investigated whether Bangladeshi children who migrate to the United Kingdom (UK) reach adrenarche earlier than non-migrants in Bangladesh or the United Kingdom.

**Methods and Findings:**

Healthy girls, aged 5–16 years, were recruited from schools in Sylhet, Bangladesh and London, England comprising four groups: Sylhetis (n = 165), first-generation migrants to the United Kingdom (n = 42), second-generation girls (n = 162), and British girls of European origin (n = 50). Anthropometric measurements were collected together with questionnaire data for migration and socioeconomic characteristics. Saliva samples were assayed for dehydroepiandrosterone (DHEAS) using enzyme-linked immunosorbent assays. Multiple linear regressions tested for group differences in anthropometric and socioeconomic variables and DHEAS levels. Median ages at adrenarche (DHEAS>400 pg/ml) were estimated using Weibull regression models for parametric survival analysis. Hazard ratios for reaching adrenarche earlier and 95% confidence intervals (CI), both unadjusted and adjusted for anthropometric variables, were estimated from the survival analyses. First-generation migrants had a median age at adrenarche (5.3 years) that was significantly earlier than Sylheti (7.2), second-generation (7.4), and European (7.1) girls. In univariate analyses, first-generation girls reached adrenarche significantly earlier than Sylhetis [HR (CI): 2.8 (1.4–5.5]. In multivariate models, first generation girls still reached adrenarche earlier than Sylhetis after adjusting for height [HR(CI): 1.9 (0.9–4.1)] and weight [HR(CI):1.7 (0.8–3.8)], but these results were attenuated.

**Conclusions:**

We suggest that rapid catch-up growth experienced by first generation girls during early childhood may explain their advanced adrenarche. The environmental conditions leading to an earlier adrenarche, as well as the health implications of this early transition, merit further exploration.

## Introduction

The adrenarcheal transition is a key early life event that marks the beginning of what is called the “juvenile” or slow-growth phase of middle childhood beginning at approximately 6–8 years of age [1]–[4]. Adrenarche is characterised by development of the zona reticularis in the adrenal cortex and the maturation of the hypothalamic-pituitary-adrenal (HPA) axis, processes which can be tracked physiologically by the steep rise in levels of dehydroepiandrosterone (DHEA) and its more ubiquitous sulfate ester, dehydroepiandrosterone sulfate (DHEAS) [5]–[7]. Adrenarche is clinically defined when serum DHEAS reaches levels>40–50 µg/dl [8]. How the initial rise and continued production of adrenal androgens are regulated is still not clearly understood, nor do we fully understand the physiological significance of adrenarche or the extent to which the timing of this process can vary. The connection between adrenarche and the development of secondary sexual characteristics led to speculation that it is a functional precursor to puberty [9]–[11]. Campbell [12], however, has recently suggested a neurological function for adrenarche in regulating the process of synaptogenesis during middle childhood when children acquire greater cognitive skills and independence.

In relation to health outcomes, clinical studies have demonstrated an earlier age at adrenarche and elevated adrenal androgens among girls born small-for-gestational-age (SGA), particularly among those who gain weight rapidly either post-natally or in early childhood, or among children who become overweight/obese during childhood. Foetal and early childhood development, and particularly the pattern of weight gain across infancy and childhood, might then influence the timing of DHEAS production and the adrenarcheal transition. SGA children with an early pattern of weight gain and earlier adrenarche are vulnerable to developing polycystic ovary syndrome (PCOS) and related metabolic disorders, including obesity, central adiposity, hypertension, insulin resistance, type 2 diabetes, and dyslipidaemia [13]–[20]. South Asians, including Bangladeshis, are also at higher risk for developing these conditions [21]–[25] giving greater urgency to understanding patterns of middle childhood development for these populations.

The period prior to adrenarche has already been speculated by Núñez-de la Mora and colleagues as a possible “critical checkpoint” for determining the trajectory of later adult reproductive development among migrant Bangladeshi women aged 18–35 years [26]. In this earlier migration study, the authors found that Bangladeshi women who had grown up in the United Kingdom (UK) and, particularly if they had migrated when aged 0–8 years, had a significantly earlier age at menarche, as well as higher ovulation rates and levels of luteal phase progesterone compared to women who still lived in Bangladesh or to women who had migrated to the UK as adults. Building upon studies that demonstrate variation in other developmental milestones such as menarche [27]–[32], we also speculate that environmental conditions experienced prior to this checkpoint would influence the timing of adrenarche itself and might accelerate the process among child migrants.

In order to continue our work attempting to elucidate the influence of the childhood environment on human reproductive characteristics, and because of the high risk for PCOS and metabolic disorders among Bangladeshi women, we initiated a subsequent migrant study of Bangladeshi children — the Adolescence among Bangladeshi and British Youth Project (ABBY) [33]. The ABBY Project studied first-generation, British-Bangladeshi child migrants and second-generation, British-Bangladeshi girls whose parents migrated from Bangladesh to the UK. We compared them to Bangladeshi girls of similar ages who continued living in Sylhet, Bangladesh, and children of European origin who attended the same UK schools as the migrant Bangladeshis. We examined levels of salivary DHEAS, anthropometrics, migration histories, socioeconomic, and other relevant characteristics in order to test the hypothesis that the timing of adrenarche occurs earlier among girls living in and migrating to resource-rich developmental environments. To test this hypothesis we made the following predictions:

Prediction 1: Girls living in the UK reach adrenarche at a younger age than girls living in Bangladesh.

Prediction 2: First-generation girls reach adrenarche at a younger age than second-generation girls and girls of European origin.

## Methods

### Subjects and Recruitment

A total of 419 healthy girls were recruited using convenience sampling from four different groups: 1) Bangladeshis living in Sylhet, Bangladesh (henceforth, referred to as “Sylhetis”, n = 165); 2) first-generation Bangladeshi migrants in the UK (“first-generation”, n = 42); 3) second-generation migrants in the UK (“second-generation”, n = 162); and 4) British girls of European origin living in the UK (“Europeans”, n = 50). We recruited girls between the ages of 5 and 16 into the study because the project was also designed to examine the timing of adrenarche in relation to menarche, which can occur as late at 16 years old. Two school settings provided a central location for data collection: a) 10 schools in East London, UK (data collected from September 2009 until December 2010), and b) 7 schools in Sylhet Town, Bangladesh (data collected from January 2011 until April 2011). London schools were chosen in areas with a high proportion of immigrants from Sylhet, Bangladesh. In Sylhet Town, we identified semi-private/semi-government schools that were likely to have pupils from middle class families because most migrants to the UK also originate from the middle class [34]. Initially, school administrators were contacted via drop-in visits and the distribution of project description materials. From this initial recruitment effort, two secondary schools enrolled in the study. Additional schools were recruited through snowballing where key contacts at the secondary school made referrals to other contacts at their feeder schools.

Figure S1 summarizes the recruitment strategy with the overall participation rate being 32%. Participation was lower in the UK (28%) compared with Bangladesh (41%), and the absolute number of first-generation girls is lower than other groups because very few children living in East London are first generation migrants themselves. Most new immigrants from Bangladesh are between the ages of 15–30 years [35] since marriage is the primary mechanism through which visas are currently issued in the UK. Many first generation migrants have children after migrating, explaining why most British-Bangladeshi children are second generation.

### Data Collection

#### Anthropometrics

Height, weight, and waist circumference were measured using standardized techniques [36] (by two researchers in the UK, and one of these (LCH) took all the measurements in Bangladesh).

#### Questionnaires

Study participants were interviewed in person by LCH or research assistants in English, Bangla, or Sylheti languages. A standardized questionnaire was used to collect information on date and place of birth, socioeconomic status, family history of migration, self-reported ethnicity, and other characteristics.

### Saliva sampling and DHEAS assays

Saliva samples for measurement of DHEAS levels were collected at the time of interview (between 09:00 and 16:00 hours) in 5 ml polystyrene tubes using gum base (Cafosa©, Barcelona, Spain) as a stimulant. Collection tubes were then placed immediately in a cooler with ice until transported to storage laboratories in either London or Sylhet. Samples were stored at −20°C and then couriered on dry ice to the Durham Ecology and Endocrinology Laboratory where they were subsequently assayed. Some samples underwent two additional freeze/thaw cycles but subsequent analyses showed no association between these freeze/thaw cycles and DHEAS levels (β = −0.14±0.12; p = 0.26). A total of 418 saliva samples were analysed by one researcher (GC) using a commercially available salivary DHEAS enzyme-linked immunosorbent assay purchased from Salimetrics© (State College, USA). The reproducibility of the assays was assessed by two pooled, blinded quality control samples in each batch. The total (within-batch and between-batch) coefficients of variation were <20%. The lower and upper limits of detection were 43 pg/ml and 16,000 pg/ml, respectively.

### Statistical Analyses

Multiple linear regression (MLR) models were used to test for differences in socioeconomic and anthropometric variables. Untransformed height, weight, body mass index (BMI; kg/m^2^), and waist circumference measurements were compared separately for girls aged <9.5 years and ≥9.5 years, where 9.5 represents the median age of all girls in the sample and when 95% of girls had reached adrenarche. Height-for-age (HAZ), weight-for-age (WAZ), BMI, and waist circumference z-scores were calculated. The MLRs tested for differences using trend tests and pairwise comparisons. Trend tests were based on a dummy variable called Residency Scale. This scale, which reflected the number of individual/ancestral generations residing in the UK, was coded 0 to 3: 0 for Sylhetis, 1 for first-generation, 2 for second-generation, and 3 for Europeans. Pairwise comparisons included each group compared to the Sylhetis, UK resident groups (first-generation, second-generation, European) collectively compared to Sylhetis, and UK-born girls (second-generation, European) collectively compared to first-generation girls.

DHEAS concentrations were log-transformed to reduce positive skewness. DHEAS levels were plotted by age for all girls. Standard MLR models were used to evaluate differences in DHEAS among groups stratified by age-quartiles and by median age of the sample. The clinical threshold for measuring the adrenarcheal transition through serum levels of DHEAS (>40–50 µg/dl) [8] was converted (400 pg/ml) to reflect concentrations found in saliva (0.1% of plasma levels) [37]. A binary variable (adrenarcheal status) was created coding all values below and above 400 pg/ml (0 and 1, respectively). Median age at adrenarche was estimated using Weibull regression models for parametric survival analysis. This model accounts for the double censoring present in the cross-sectional data collected here; i.e., some of the girls had not yet reached adrenarche at their current age (right-censored) or had reached adrenarche at some unknown age in the past (left-censored). The median ages at adrenarche for each migration group were then derived from the Weibull regression models. Weibull regression models were also used to create plots for the distribution density of age at onset of adrenarche for each migration group. Age at adrenarche was initially modelled using all girls combined and then restricted to girls aged <9.5 years. Models adjusted for height-for-age (HAZ), weight-for-age (WAZ), BMI-for-age (BMI-Z), and waist circumference-for-age (WCA-Z) z-scores were restricted to girls aged <9.5 years since older girls are at least three years post-adrenarche.

To test the first prediction, we compared the UK resident groups individually and collectively to Sylheti girls and tested for a trend using the Residency Scale. To test the second prediction, first-generation girls were compared to all UK-born girls. All statistical analyses were conducted using Stata Version 11, and statistical significance was set at p<0.05.

### Ethics Committee Approval

The ABBY Project received ethical permission from the Department of Anthropology, Durham University Ethics Committee and the MAG Osmani Medical College, Sylhet, Bangladesh (#2443-2). The Office of Human Research Subjects at the National Cancer Institute issued an Institutional Review Board exemption based on the existing approvals. Written, informed consent was provided by parents of the participants and written, informed assent was provided by the participants. The data are available upon request through a data transfer agreement through the Intramural Division of the National Cancer Institute.

## Results

Among Sylheti girls, 50% had relatives living in the UK. The mean age of arrival in the UK for first-generation, British-Bangladeshi girls was 4.8±4.2 (SD) years, with a range from 0 to 15; the median age was 4.0 years. Families of girls in the UK had lower employment rates but higher educational qualifications than families of girls living in Bangladesh. Fathers of first-generation girls had a slightly lower employment rate (67%) compared to fathers of either second-generation (77%) or European (76%) girls, but this difference is not significant (p = 0.2). None of the mothers from any of the Bangladeshi groups [Sylhetis (19%), first- (21%) or second-generation (26%)] had a high employment rate, but almost half of mothers of European girls in London were employed (44%). Parents of first-generation girls were less likely to own a house compared to the parents of Sylheti girls in Bangladesh or second-generation girls, but were more likely than parents of European girls (Table 1).

**Table 1:** Sample characteristics of ABBY project participants; reported as mean (SD) for continuous variables or number and percent for categorical variables.

	Sylheti		First-Generation		Second-Generation		European		P for trend; Residency Scale	Pairwise comparison Sylheti vs. UK resident	Pairwise comparison First-generation vs. UK born
**N**	165		42		162		50				
Age (years)	9.7	(3.0)	10.5†	(3.4)	10.1	(2.7)	9.7	(2.5)	0.13	0.16	0.14
**Socioeconomic Variables**											
** Household**											
Household size (people)	6.6	(2.6)	5.8†	(2.2)	5.9†	(1.7)	4.2†	(1.3)	<0.01	<0.01	0.70
Family owns house	100	52%	8†	23%	51	42%	7†	19%	<0.01	<0.01	0.06
Family owns a car	29	17%	20†	50%	109†	71%	28†	57%	<0.01	<0.01	0.01
Single Mother Household	30	17%	2	5%	22	18%	19†	44%	<0.01	0.10	0.00
** Employment**											
Father (in households with both parents)	133	94%	22†	67%	80†	77%	16†	76%	<0.01	<0.01	0.23
Mother	33	19%	8	21%	39†	26%	20†	44%	0.04	0.01	0.14
** Education (college or higher)**											
Father (in households with both parents)	29	32%	16†	57%	35	45%	10†	59%	0.02	0.05	0.50
Mother	18	16%	13	39%	56	42%	25	76%	<0.01	<0.01	0.33
**Anthropometrics**											
** Height (cm)**											
height for age z-score	−1.2	(1.3)	−0.8†	(1.0)	−0.2†	(1.3)	0.1†	(1.2)	<0.01	<0.01	0.01
<9.5 yr.	116.4	(9.8)	123.6†	(8.9)	124.8†	(9.9)	126.5†	(7.9)	<0.01	<0.01	0.64
>9.5 yr.	144	(11.3)	148.7	(7.7)	147.9	(9.8)	144.9	(11.8)	0.24	0.08	0.63
** Weight (kg)**											
weight for age z-score	−1.2	(1.6)	0.1†	(1.4)	0.3†	(1.3)	0.8†	(1.2)	<0.01	<0.01	0.17
<9.5 yr.	20.5	(5.8)	28.4†	(10.0)	28†	(8.3)	29.6†	(6.4)	<0.01	<0.01	0.69
>9.5 yr.	37.8	(10.6)	47.1†	(10.2)	45.9†	(12.3)	44.9†	(13.8)	<0.01	<0.01	0.70
** Waist Circumference (cm)**											
waist for age z-score	−1.8	(9.3)	0.7†	(1.5)	1.0†	(1.2)	1.4†	(1.0)	<0.01	<0.01	0.08
<9.5 yr.	48.9	(6.4)	59.8†	(9.4)	58.7†	(7.4)	61†	(5.4)	<0.01	<0.01	0.76
>9.5 yr.	58.3	(6.9)	65.8†	(8.6)	67.8†	(9.3)	68.1†	(9.1)	<0.01	<0.01	0.35
** BMI (kg/m^2^)**											
BMI for age z-score	−0.6	(1.3)	0.7†	(1.4)	0.6†	(1.2)	1.0†	(1.1)	<0.01	<0.01	0.89
<9.5 yr.	15.0	(3.1)	18.6†	(4.3)	17.7†	(3.2)	18.5†	(2.5)	<0.01	<0.01	0.39
>9.5 yr.	18.0	(3.1)	21.2†	(3.6)	20.5†	(3.6)	21.4†	(4.0)	<0.01	<0.01	0.57

† indicates statistical difference between Sylheti and the other group at the p<0.05 cut off.

**Figure 1:**
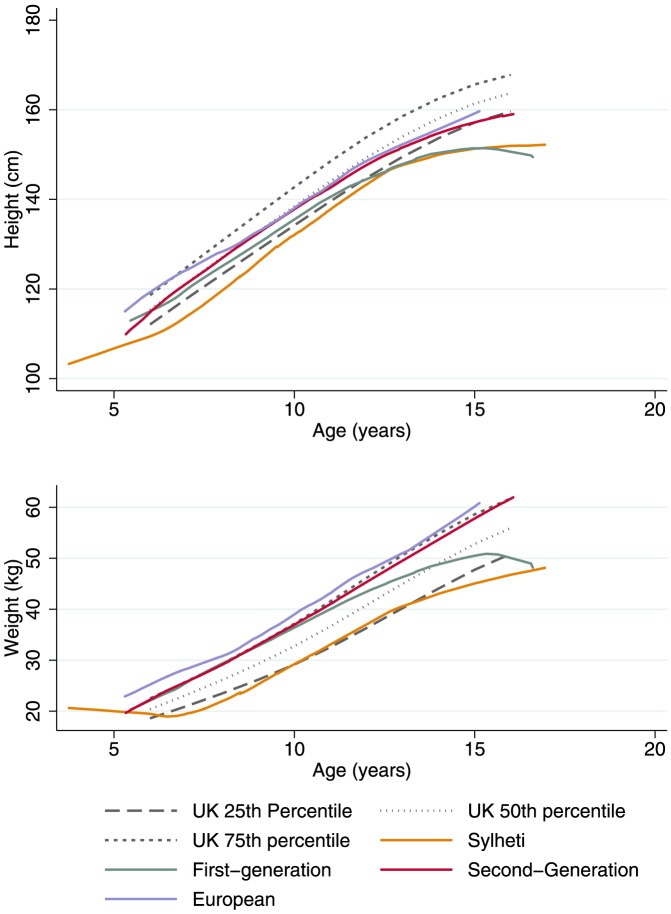
Height and Weight by among girls aged 5–6 in the ABBY Project. (Upper) Lowess smoother of height and age for each group compared with the UK 1990 Growth References, (Lower) Lowess smoother of weight and age for each group compared with the UK 1990 Growth References.

Figure 1 shows the relationships between height/weight and age for each of the groups with reference to the UK 1990 growth standards [38]. All groups of girls residing in the UK were significantly heavier (WAZ: first-generation  = 0.1; second-generation  = 0.3; Europeans  = 0.8) and taller (HAZ: first-generation  = -0.8; second-generation  = -0.2; Europeans  = 0.1) than the girls living in Bangladesh (WAZ = -1.2; HAZ = −1.2; all p<0.05) (Table 1). The BMI-Z of first-generation girls (0.7) is similar to the second-generation (0.6) and to the European girls (1) (p = 0.9).

**Figure 2:**
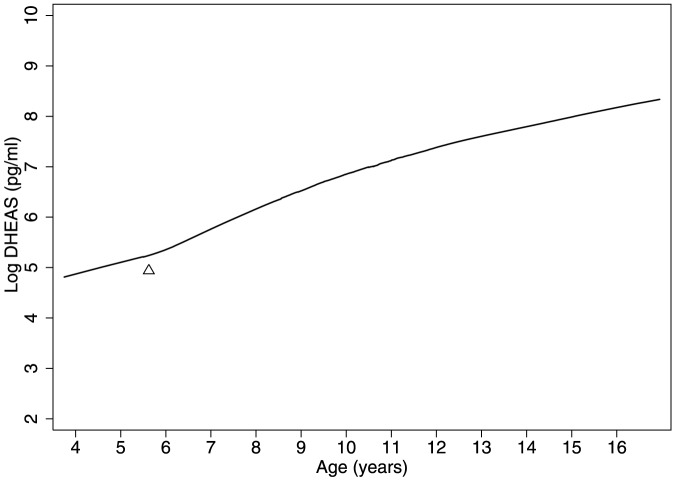
Lowess smoother of the log of DHEAS by age among all girls Aged 5–16 years. Triangle shows inflection point where log salivary DHEAS reaches 5.5–6 pg/ml, signifying the clinical threshold for adrenarche.

**Figure 3:**
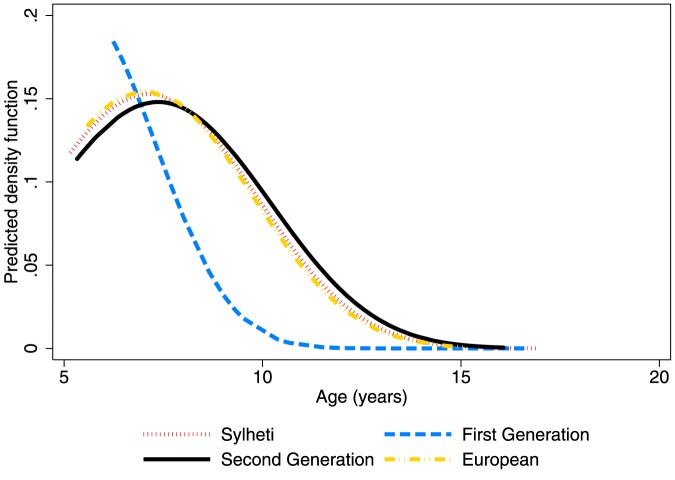
Comparison of the distribution density of the age at onset of adrenarche among Sylheti, first-generation, second-generation, and European girls determined by Weibull regression models for parametric survival analysis. The curves plot the predicted density of adrenarche on the Y axis and by age on the X axis, with the peak of each curve corresponding to the median age at adrenarche. The median age at adrenarche for each migration group was: Sylheti = 7.2, first-generation = 5.3, second-generation = 7.4, Europeans = 7.1; p-trend = 0.7. First-generation girls reached adrenarche significantly earlier than all other groups (p<0.01).

Table 2 shows mean levels of salivary DHEAS for each group by age quartiles. Salivary DHEAS levels increased with age in all groups as expected. Levels were slightly higher among the groups of girls <9.5 years living in the UK (6.1±0.2; p = 0.06), and highest among first-generation girls (6.4±1.0; p = 0.05), compared with those living in Bangladesh (5.8±1.4). There were no differences in age-adjusted DHEAS levels by age at migration (p = 0.7) or time since migration (p = 0.6). A lowess smoother curve of DHEAS by age shows an inflection in levels where log DHEAS reaches 5.5–6 pg/ml (Figure 2) corresponding to the clinical cut point of 400 pg/ml of DHEAS (5.9 on the log scale) [8]. Median age at adrenarche was calculated for girls aged <9.5 years and occurred significantly earlier in first-generation migrants (5.3 years) compared to second-generation (7.4 years), European (7.1 years), and Sylheti (7.2 years) girls (p<0.01, Figure 3). The corresponding hazard ratios (95% CI) for reaching adrenarche are: Sylheti = 1, first-generation = 2.6 (1.3–5.2), second-generation  = 1.0 (0.6–1.5), European  = 1.0 (0.5–1.9) (Table 3). The earlier adrenarche in first-generation girls compared to Sylheti girls was attenuated and lost significance after individually adjusting for z-scores of height, weight, BMI, and waist circumference and also after adjusting for all anthropometrics simultaneously (Table 3). The difference in age at adrenarche between first-generation girls and UK-born girls remained significant after adjustment, individually and collectively, for each anthropometric variable [all p<0.05; Table 3]. Time of day of collection did not affect results (data not shown).

**Table 2:** Salivary DHEAS pg/ml by age groups in Sylheti, first-generation, second-generation and European girls, aged 5-16 years.

Variables	Sylheti			First Generation			Second Generation			European			P for trend; Residency Scale	Pairwise comparison Sylheti vs. UK residents	Pairwise comparison First-generation vs. UK born	
	(n = 165)			(n = 42)			(n = 162)			(n = 50)						
	*n*	Mean	SD	*N*	Mean	SD	*n*	Mean	SD	*n*	Mean	SD				
Age Quartile																
1 (<7.5 yr.)	45	5.3	(1.4)	11	5.98	(0.8)	30	5.78	(1.2)	8	5.41	(0.7)	0.23	0.07	0.77	
2 (7.5–<9.5 yr.)	46	6.22	(1.2)	8	6.95	(1.1)	44	6.36	(1.1)	15	6.39	(1.0)	0.42	0.32	0.93	
3 (9.5–<11.8 yr.)	36	7.28	(0.7)	4	6.89	(0.9)	44	7.14	(1.0)	16	7.25	(1.0)	0.79	0.48	0.28	
4 (>11.8 yr.)	38	7.82	(0.8)	19	7.57	(0.8)	44	7.76	(0.9)	11	7.67	(0.9)	0.75	0.48	0.23	
<9.5 yr.	91	5.8	(1.4)	19	6.4†	(1.0)	74	6.1	(1.2)	23	6	(1.0)	0.12	0.06	0.31	
≥9.5 yr.	74	7.6	(0.8)	23	7.4	(0.8)	88	7.5	(1.0)	27	7.4	(1.0)	0.85	0.40	0.98	

† indicates statistical difference between Sylheti and first-generation girls at the p<0.05 cut off.

**Table 3:** Hazard ratios (95% C I) of adrenarche onset among girls <9.5 years adjusted for height, weight, BMI, and waist circumference.

Adrenarche	HR	95% CI		p-value	p for trend	Pairwise comparison Sylheti vs. UK residents	Pairwise comparison First-generation vs. UK born
Unadjusted							
Anthropometrics							
Height for Age Z-score	1.2	(1.1- 1.4)		0.002			
Weight for Age Z-score	1.2	(1.0-1.3)		0.015			
BMI Z-score	1.1	(1.0-1.2)		0.198			
Waist Circumference	1.0	(1.0-1.1)		0.034			
Group							
Sylheti	1.0						
First-generation	2.6	(1.3-5.2)		0.006			
Second-generation	1.0	(0.6-1.5)		0.856			
European	1.0	(0.5-1.9)		0.998			
Group Differences					0.83	0.56	0.01
Group differences adjusted for anthropometrics							
Height for Age Z-score	1.3	(1.1-1.5)		0.159			
Sylheti	1.0						
First-generation	1.9	(0.9-4.1)		0.105			
Second-generation	0.6	(0.4-1.1)		0.098			
European	0.7	(0.3-1.4)		0.255			
Group Differences					0.08	0.29	0.01
Weight for Age Z-score	1.2	(1.1-1.4)		0.005			
Sylheti	1.0						
First-generation	1.7	(0.8-3.8)		0.159			
Second-generation	0.7	(0.4-1.1)		0.109			
European	0.7	(0.3-1.4)		0.290			
Group Differences					0.09	0.28	0.02
BMI Z-Score	1.1	(1.0-1.3)		0.187			
Sylheti	1.0						
First-generation	2.0	(0.9-4.2)		0.073			
Second-generation	0.8	(0.5-1.3)		0.394			
European	0.9	(0.4-1.8)		0.709			
Group Differences					0.36	0.78	0.02
Waist Circumference	1.0	(1.0-1.1)		0.047			
Sylheti	1.0						
First-generation	1.8	(0.8-4.1)		0.131			
Second-generation	0.8	(0.4-1.3)		0.331			
European	0.9	(0.4-1.9)		0.701			
Group Differences					0.31	0.64	0.04
Height, Weight, BMI, and Waist							
Sylheti	1.0						
First-generation	1.8	(0.8-4.0)		0.180			
Second-generation	0.7	(0.4-1.2)		0.164			
European	0.7	(0.3-1.5)		0.363			
Group Differences					0.12	0.35	0.03

## Discussion

This paper tested the hypothesis that age at adrenarche will vary depending on the environment of development using two predictions: first, that girls living in London would reach adrenarche at earlier ages compared to girls living in Bangladesh and, secondly, that first-generation migrants would reach adrenarche earlier than other groups resident in London regardless of Bangladeshi or European origin. Only the second hypothesis was fully supported.

In relation to the first prediction, the median age at adrenarche did not differ significantly among Sylheti girls, second-generation migrants, or girls of European origin, but first generation girls had an earlier median age at adrenarche than Sylhetis. Greater height, weight, and BMI partially explained the earlier age at adrenarche among first-generation girls. In relation to the second prediction, first-generation girls reached a median age at adrenarche two years earlier than the other UK groups, a difference that was statistically significant independent of differences in growth.

Salivary DHEAS levels were highest among the first-generation, British-Bangladeshi girls aged <9.5 years. However, after age 9.5 years, first-generation girls had the lowest levels of DHEAS and Sylhetis had the highest, with second-generation and European girls having intermediate levels. In a previous study measuring androgens among American girls of different ethnicities, African-American girls had higher levels of androgens before age 10, but not at older ages [39]. It has been suggested that once puberty begins, adrenal androgen levels are tempered by other hormonal changes [40].

The mechanisms that govern onset of the adrenarcheal transition are still poorly understood by researchers. However, documented variation in levels of DHEAS within clinical populations suggests that the age at which girls reach adrenarche is variable [41]–[45] and is likely related to a complicated signalling pathway involving several metabolic and regulatory hormones involved in growth and development. Two factors of growth and development appear to be key: size at birth and patterns of weight gain in childhood, including catch-up growth. Precocious adrenarche, frequently diagnosed and defined by the early appearance of pubic hair before the age of 8 years, is often associated with SGA children, but particularly in children who gain weight rapidly in early childhood [15], [16], [19], [46]. We have no data on birth size for girls in the study here, and there is no clinical evidence that the first-generation British-Bangladeshi girls experience “precocious” adrenarche. However, South Asian groups in the UK are generally characterized by lower average infant birth weights and greater fat mass, and these characteristics appear, so far, to be unchanging across generations [47]–[49]. Mean birth weight for Bangladeshi infants born in the UK is just over 3000 g, an average 250 g lighter than offspring born to white UK women [47], [48]. This average is also 500 g higher than the <2500 g cut-off for low birth weight (LBW) babies. Despite lower birth weights for Bangladeshi infants in the UK relative to white infants, migrant mothers may be delivering heavier infants compared to those born in Bangladesh. One of the highest published mean birth weights for infants born to an urban population of Bangladeshi mothers attending a government hospital in Dhaka was 2889±468 g, but this study included women from different socioeconomic groups [50]. Mean birth weight for infants of affluent families in a UNICEF-sponsored survey of selected areas of Bangladesh was still only 2732±440 g [51]. The mean proportion of LBW babies even among highly educated, affluent women in Dhaka was 15% [52]. It is therefore possible that a higher proportion of the first-generation girls who were born in Bangladesh could have been LBW compared to the proportion among the Bangladeshi and European girls born in the UK.

The heights and weights of first-generation girls measured in this study are, however, similar to those of UK-born girls — between the 50–75^th^ percentiles of UK growth standards — while the Sylheti girls hover around the 25^th^ percentile. Data from an earlier study of affluent Dhaka children also show that heights and weights were also only around the 25^th^ percentile of the United States National Center for Health Statistics' standards [53]. The data presented here for migrants mirror other UK findings where the growth of South Asian infants and children have become comparable to 1990 growth standards for white UK children [54], and also for earlier 1970 standards [55]. In addition, there is a secular trend towards increased height in successive generations of UK South Asians, especially females [56] despite the continued trend for smaller neonates relative to the white UK population. All these data strongly support the idea that first-generation girls experience a period of rapid catch-up growth following migration.

Rapid catch-up growth and weight gain in early childhood among first-generation girls after migrating to the UK could explain their earlier transition to adrenarche. Children from the ALSPAC longitudinal cohort who gave blood samples at the age of eight years had significantly higher levels of DHEAS if they had been heavier relative to other children between the ages of 0–3. A combination of low birth weight and higher weight when the ALSPAC children were sampled also independently predicted levels of DHEAS [43]. Similarly, girls who are SGA and experience subsequent catch-up growth are more likely to have a premature adrenarche [13]–[15], [20]. The fact that the difference in age at adrenarche was no longer significant between first-generation and Sylheti girls after controlling for anthropometric variables, but was still significant between first-generation and UK-born girls, suggests that catch-up growth specifically, rather than larger body size, may explain the variation in adrenarcheal timing.

Catch-up growth has already been suggested as a mechanism to explain earlier *pubertal* development, including menarcheal age, among migrant adoptees in developed countries who are often shorter and thinner on arrival than girls in the host country [28], [57]–[60]. Even among non-migrants, LBW girls who then gain weight rapidly relative to their peers have the earliest ages at menarche compared to peers whose weight remains stable or even compared to girls born with normal birth weights who also gain weight [61]. Unfortunately, the physiological mechanisms that might influence this pubertal transition remain unknown [62], although Teilmann et al. [59] have suggested that it is centrally driven given higher levels of reproductive hormones in pre-pubertal adopted girls. Other suggested mechanisms include: increased metabolic activity that would accelerate linear growth initiating an earlier puberty [63], hyperplasia of adipose tissue with increasing fatness that could influence aromatisation of androgens to oestrogens and trigger pubertal development [64], stimulation of IGF-1 secretion following an increase in dietary protein intake [60], or psychosocial stress associated with disrupted emotional development in early life [65].

The rise in adrenal androgens that marks adrenarche in normal, healthy children coincides with the adiposity rebound when the number and size of adipocytes begin to increase following a childhood nadir in BMI. This occurs at approximately ages 5–6 years, but can vary enormously from around 1.5 years to age 8 [64], [66]–[68]. There is evidence that a period of rapid catch-up growth among children could lead to an earlier adiposity rebound independent of birth weight [69]. An earlier adiposity rebound is also linked to higher BMIs in adulthood, particularly when the individuals had a low fat mass during infancy [64], [67], [70], findings which are similar to SGA infants who experience rapid post-natal catch-up growth. However, more recently, Rolland-Cachera et al. noted that only rapid post-natal weight gain rather than a low birth weight predicted an earlier adiposity rebound [69]. In their longitudinal study of urinary DHEAS in pre- and post-pubertal children, Remer and Manz noticed that increases in median DHEAS levels matched the largest change in BMI during childhood, indicating an association between fatness and adrenarche [42]. They suggest that a marked change in nutritional status could serve to regulate the timing of adrenarche. It is therefore possible that rapid catch-up growth could serve as this kind of marked change. The gradual increase in fatness that signals the adiposity rebound may represent the normal, age-related “trigger” for increases in IGF-1, insulin, leptin, and other hormones during childhood. Each of these has been linked to stimulation of cytochrome P450c17 resulting in the adrenarcheal rise in DHEA and DHEAS, although contradictory studies at present cloud a full understanding of the relationships involved [71]–[75]. In first-generation, British-Bangladeshi girls, an earlier change in nutritional status following migration could interfere with the usual timing of this adiposity rebound. Accelerated weight and height velocities could result in earlier adiposity signals that could advance the adrenarcheal transition.

Changes in energetic status for first-generation migrants are probably due to a number of factors, and we should emphasize that Bangladeshi migrants to the UK originate from relatively affluent middle-class families who are not nutritionally stressed. Migration to the UK, however, leads to a reduction in infectious and parasitic diseases that might translate into higher energy availability for growth. In our studies of older Bangladeshi women aged 35–59, exposure to infectious and parasitic diseases that carry heavy immunological and energetic costs across the lifespan were significant predictors of earlier reproductive ageing among Sylhetis and adult migrants [76], [77]. There are also qualitative differences in nutritional intake in the UK after migration even though many migrants maintain their traditional diets and can purchase most of what they need, including local Bangladeshi foods, from London stores, [78], [79]. Migrants eat less rice and curry [33], more junk food, and have a higher protein intake [80]. Rolland-Cachera et al. have discussed how a change in infant diets in industrialised countries from high fat/low protein to low fat/high protein may have affected linear growth across childhood and predisposed children to an earlier adiposity rebound [81]. Migrant Bangladeshi women in the UK also rapidly reduce the frequency and prevalence of breastfeeding compared to the counterparts who remain in Sylhet [82], reinforcing the possibility that dietary quality is changing for their infants. First-generation children then might encounter a series of rapid changes to their energetic status that could explain the acceleration in weights and heights and the significantly earlier age at adrenarche for this group. However, catch-up growth does not explain all the variation in adrenarcheal timing; other factors that accompany migration may also be implicated. Psychosocial stress that accompanies migration may have some impact on developmental schedules among first-generation immigrants [65].

It is unlikely that first-generation migrants were self-selected for larger size prior to migration given the lower socioeconomic family indicators for this group compared to either Sylhetis or second-generation families. For many socioeconomic and educational variables, Bangladeshi residents in London were more comparable to the European families than they were to their counterparts still living in Bangladesh, partly reflecting similarities in neighbourhood socioeconomic statistics in school districts from which the children were recruited. However, families of first generation girls were worse off in terms of their parents' employment. These socioeconomic differences may arise from the difficulties of transitioning to the UK economy given that many first-generation parents lack fluency in English and may not have work skills that translate easily into employment. The higher rates of employment for European mothers may reflect cultural preferences that influence Bangladeshi women to remain at home rather than any contrast in qualifications. The differences in socioeconomic variables between first-generation girls and the other groups most likely reflect economic changes that accompany migration, rather than a selection bias.

Children who experience an earlier adiposity rebound, and particularly where they were either SGA or LBW and gained weight rapidly in infancy, are generally at higher risk for central adiposity, overweight, and obesity as adults [41], [64], [67]. They are also at risk for related metabolic disorders including polycystic ovary syndrome, hypertension, insulin resistance, type 2 diabetes, and dyslipidaemia [13]–[15], [20], [43]. One of the actions of DHEAS is to stimulate the uptake of glucose by adipose cells and this is most marked in sites closely adjacent to the zona reticularis, thereby encouraging abdominal fat deposition [12]. This tendency might also explain why children with precocious adrenarche are characterised by an increase in central adiposity and are at greater risk for developing metabolic disorders, conditions to which South Asians, including Bangladeshis, are particularly susceptible [23], [24], [83], [84]. Further research on these conditions in relation to early life development among South Asians is warranted.

### Study limitations

The study is cross-sectional and girls were not followed longitudinally during childhood as they made the transition to adrenarche. Variation across groups may therefore reflect potential differences in age-cohorts through time. However, the migrant study design offers a novel perspective on childhood development. Few studies compare groups of the same ethnicity in terms of two generations and two locations and compare them with populations in both the “home” and “host” countries. The sample size for the first-generation girls is lower than samples for the other groups given the smaller numbers of recent migrants in UK schools relative to UK-born children. This discrepancy may affect the statistical calculations. Small samples sizes within the first generation girls also affected our ability to look at differences in adrenarcheal timing in relation to age and time since migration. ABBY studied healthy volunteers and, due to ethical considerations in collecting blood samples from children, we have no measurements for circulating levels of IGF-1, insulin, leptin, or genetic markers that might help in our interpretation of the adrenarcheal transition.

## Conclusions

This study follows earlier findings where first-generation Bangladeshi women of prime reproductive age (18–35 years) who had moved to UK as children had an earlier self-reported age at menarche, significantly higher levels of salivary progesterone and rates of ovulation as adults compared with migrants who had migrated as adults. We had speculated from these findings that the childhood environment prior to adrenarche might represent a key period influencing adult reproductive function, and that adrenarche itself might represent a critical childhood threshold for later life development. The study of Bangladeshi migrant children aged 5–16 years presented here supports this contention. First-generation Bangladeshi girls who moved to the UK experience adrenarche two years earlier than Sylhetis who remain in Bangladesh, as well as second-generation British-Bangladeshi girls and girls of European origin. We suggest that rapid catch-up growth experienced by first-generation girls during early childhood may explain their accelerated adrenarche, and that an earlier adiposity rebound provides the stimulus for development of the zona reticularis and production of DHEAS. Rapid catch-up growth may occur in response to reductions in pathogen loads and access to better quality health care following relocation to the UK rather than major changes in dietary quality or quantity although these relationships need greater elucidation. The relationship between an earlier adrenarche and subsequent pubertal maturation remains to be further explored in this population as do the potential health risks in adulthood from an earlier childhood maturation.

## Supporting Information

Figure S1 **Flow chart of school and participant recruitment.** Recruitment, via convenience sampling, commenced in September 2009 in London, England and completed in Sylhet, Bangladesh in April 2011 resulting in the enrolment of 17 schools and 488 participants. Complete data were available for 419 participants.
(EPS)
